# Characterization of the Powdery Mildew Resistance Gene in the Elite Wheat Cultivar Jimai 23 and Its Application in Marker-Assisted Selection

**DOI:** 10.3389/fgene.2020.00241

**Published:** 2020-04-02

**Authors:** Mengshu Jia, Hongxing Xu, Cheng Liu, Ruixi Mao, Haosheng Li, Jianjun Liu, Wenxiao Du, Wenrui Wang, Xu Zhang, Ran Han, Xiaolu Wang, Liru Wu, Xiao Liang, Jiancheng Song, Huagang He, Pengtao Ma

**Affiliations:** ^1^School of Life Sciences, Yantai University, Yantai, China; ^2^State Key Laboratory of Crop Stress Adaptation and Improvement, School of Life Sciences, Henan University, Kaifeng, China; ^3^Crop Research Institute, Shandong Academy of Agricultural Sciences, Jinan, China; ^4^Shandong Seed Administration Station, Jinan, China; ^5^School of Food and Biological Engineering, Jiangsu University, Zhenjiang, China

**Keywords:** wheat powdery mildew, *PmJM23*, BSR-Seq, marker-assisted selection, agronomic trait

## Abstract

Powdery mildew infection of wheat (*Triticum aestivum* L.), caused by *Blumeria graminis* f. sp. *tritici* (*Bgt*), is a destructive disease that threatens yield and quality worldwide. The most effective and preferred means for the control of the disease is to identify broad-spectrum resistance genes for breeding, especially the genes derived from elite cultivars that exhibit desirable agronomic traits. Jimai 23 is a Chinese wheat cultivar with superior agronomic performance, high-quality characteristics, and effective resistance to powdery mildew at all growth stages. Genetic analysis indicated that powdery mildew resistance in Jimai 23 was mediated by a single dominant gene, tentatively designated *PmJM23*. Using bulked segregant RNA-Seq (BSR-Seq), a series of markers was developed and used to map *PmJM23. PmJM23* was then located at the *Pm2* locus on the short arm of chromosome 5D (5DS). Resistance spectrum analysis demonstrated that *PmJM23* provided a broad resistance spectrum different from that of the documented *Pm2* alleles, indicating that *PmJM23* is most likely a new allele of *Pm2*. In view of these combined agronomic, quality, and resistance findings, *PmJM23* is expected to be a valuable resistance gene in wheat breeding. To efficiently use *PmJM23* in breeding, the closely linked markers of *PmJM23* were evaluated and confirmed to be applicable for marker-assisted selection (MAS). Using these markers, a series of resistant breeding lines with high resistance and desirable agronomic performance was selected from the crosses involving *PmJM23*, resulting in improved powdery mildew resistance of these lines.

## Introduction

Common wheat (*Triticum aestivum* L.) is one of the three major grain crops worldwide, and its high and stable yield plays an important role in food security. However, various diseases, including powdery mildew, rusts, and fusarium head blight, can have devastating impacts on yield (Huang and Pang, 2017; Ingvardsen et al., 2019; Li et al., 2019b). Powdery mildew caused by *Blumeria graminis* f. sp. *tritici* (*Bgt*) is one of the most damaging diseases, typically decreasing wheat yield by 10–15% and up to 50% in severe cases (Morgounov et al., 2012; Xu et al., 2015). In China alone, the area of winter wheat affected annually by powdery mildew has exceeded 6 m ha in recent decades, causing 300,000 tons of crop loss each year^1^.

Although the pesticides are commonly used for powdery mildew control, resistance to drugs and environmental pollution have become increasingly prominent (de Waard et al., 1986; Felsenstein et al., 2010). Improved host resistance provides an attractive opportunity for the development of an effective and environmentally acceptable means to control this disease (Ma et al., 2015a, 2018, 2019). In wheat production, the powdery mildew resistance (*Pm*) genes exhibit mainly race-specific resistance, which was often short lived, as they were defeated by the fast-evolving virulent pathogen (Xiao et al., 2013; El-Shamy et al., 2016). The ratio of broad-spectrum resistance in Chinese wheat cultivars/breeding lines is not yet satisfactory in wheat production (Li et al., 2011). Therefore, there is an urgent need to mine and utilize more effective resistance sources to increase the genetic diversity of *Pm* genes.

To date, more than 70 *Pm* genes/alleles (*Pm1–Pm65*, *Pm8* is allelic to *Pm17*, *Pm18* = *Pm1c*, *Pm22* = *Pm1e*, *Pm23* = *Pm4c*, *Pm31* = *Pm21*) have been identified in 60 loci from common wheat and its relatives (Li et al., 2019a; McIntosh et al., 2019). However, not all the genes can be directly used in resistance breeding. Many *Pm* genes have adverse pleiotropism, linkage drag, or competition lag due to their genetic characteristics. For example, the gene *Pm16* has broad-spectrum resistance to wheat powdery mildew, but the linkage drag associated with *Pm16* leads to a 15% yield loss (Summers and Brown, 2013). The gene *Pm8* derived from the 1RS chromosome of rye made a significant contribution to the control of wheat powdery mildew in 1990s, but the linked secalin glycopeptide in 1RS resulted in a decline in flour quality (Friebe et al., 1989; Lee et al., 1995). Additionally, the *Pm* genes derived from landraces usually have poor agronomic performance and need multigeneration backcrossing before acceptance by breeders (Xu et al., 2015; Li et al., 2020). Clearly, the value to breeding of a specific *Pm* gene depends not only on its effectiveness for disease control but also on the agronomic performance of its donor (Zhao et al., 2013; Ma et al., 2018). The discovery of new genes or new allelic variations from elite wheat cultivars offers an attractive prospect for the rapid genetic improvement of resistance.

While conventional wheat breeding has been remarkably successful in many respects, it is usually subjective, inefficient, and unable to achieve stable improvement (Gupta et al., 2010). Molecular breeding programs worldwide can successfully provide a valuable complement to conventional breeding (Kuchel et al., 2007; Gupta et al., 2010). With the development of molecular markers, marker-assisted selection (MAS) can facilitate the exclusion of adverse genes in fewer generations and accelerate breeding progress (Jiang et al., 2017). Techniques including high-throughput single-nucleotide polymorphism (SNP) arrays, specific-locus amplified fragment sequencing (SLAF-Seq), and bulked segregant RNA-Seq (BSR-Seq) especially have been widely used for genetic mapping, thereby accelerating the cloning and utilization of superior wheat genes (Liu et al., 2018; Wu et al., 2018; Shi et al., 2019; Tan et al., 2019). Furthermore, advances continue to be made in whole genome sequencing of wheat. Common wheat (AABBDD) and its diploid (AA and DD genomes) and tetraploid (AABB) ancestors all have relatively perfect genome sequences (Avni et al., 2017; Luo et al., 2017; The International Wheat Genome Sequencing Consortium, 2018; Ling et al., 2018), which will greatly facilitate the effectiveness of SNP chip, BSR, and SLAF data in cloning of resistance genes and their utilization in breeding.

Jimai 23 is an elite wheat cultivar developed by the Shandong Academy of Agricultural Sciences (China) derived from Jimai 22, which is the most widely grown wheat cultivar in China during the last decade. Previous reports using a single *Bgt* isolate demonstrated that the powdery mildew resistance in Jimai 22 is controlled by a single dominant gene, *Pm52* (Yin et al., 2009; Qu et al., 2019). In recent years, Jimai 23 also showed highly effective resistance to powdery mildew, indicating that it is an attractive source for controlling wheat powdery mildew. To clarify the relationship between the *Pm* genes in Jimai 23 and 22 and to better use the powdery mildew resistance in Jimai 23, we report, in this work, the identification and dissection of the *Pm* gene(s) in Jimai 23, development of molecular markers for, and breeding with, the *Pm* gene(s) in Jimai 23.

## Materials and Methods

### Plant Materials

The wheat cultivar Jimai 23 was bred from the cross of Jimai 22 and Yumai 34 by the Crop Research Institute, Shandong Academy of Agricultural Sciences, and used as the donor of resistant gene(s) against powdery mildew in this research. The wheat cultivar Tainong 18 was used as a susceptible parent and crossed with Jimai 23 to obtain an F_2_ population and F_2__:__3_ families for genetic analysis of powdery mildew resistance in Jimai 23. Wheat cultivars Huixianhong and Mingxian 169, which were susceptible to all the *Bgt* isolates tested, were used as the susceptible controls for phenotypic assessment. Eight resistant donors with documented *Pm* genes (Supplementary Table S1) were used to compare their phenotypic responses to different *Bgt* isolates with those of Jimai 23 and to evaluate the breeding value of *Pm* gene(s) in Jimai 23 through resistance spectrum analysis. Jimai 22, one of the parents of Jimai 23, is a super-high yield and medium gluten wheat cultivar showing good resistance to wheat powdery mildew and stripe rust (Yin et al., 2009; Chen et al., 2016; Qu et al., 2019). In this study, Jimai 22 was also used as the control for evaluation of comprehensive traits of Jimai 23. Liangxing 99 with documented *Pm52* (Zhao et al., 2013) was used for comparing the relationship of its *Bgt* resistance gene with that in Jimai 22. Twenty-six susceptible wheat cultivars from different ecological regions of China were used to evaluate the availability of closely linked markers for MAS, five of which (Gaoyou 5766, SH4300, Tainong 2419, 125574, and Daimai 1503) had been crossed with Jimai 23 for MAS (Supplementary Table S2).

### Evaluation of Comprehensive Traits

From 2012 to 2016, Jimai 23 and 22 were planted in the field at the Crop Research Institute, Shandong Academy of Agricultural Sciences (Jinan, China), for a comprehensive evaluation of their traits. Sowing and assessment were based on the methods of Xu et al. (2014). Seeds of Jimai 23 and 22 were sown in six rows (3.0 m in length and inter-row distance of 20 cm) with two rows of susceptible controls as guard rows on each side of the plot. Spike numbers per mu (SNM) (1 ha = 15 mu), kernel numbers per spike (KNS), thousand kernel weight (TKW), yield per mu (YM) (1 ha = 15 mu), bulk density (BD), gluten index (GI), sedimentation value (SV), and stable time of dough (STD) were analyzed to evaluate comprehensive traits of Jimai 23 using Jimai 22 as control. The method for assessing agronomic and yield traits, such as SNM, KNS, YM, and BD, are described in Teich (1984) and Xu et al. (2014), and grain quality traits, such as GI, SV, and STD, are described in Studnicki et al. (2018) and Kuchel et al. (2006). In each year, three replicates were sampled using the same procedure to confirm the phenotypic data. Analysis of variance (ANOVA) of each trait was performed using SPSS 16.0 software (SPSS Inc., Chicago, IL, United States), with a significance level of *p* ≤ 0.05.

### Phenotypic Evaluation of Reactions to Different *Bgt* Isolates

From 2017 to 2019, Jimai 23 and eight resistant donors with documented *Pm* genes (Supplementary Table S1) were planted in the greenhouse at Yantai University (Yantai, China) for disease assessment at the adult stage. They were planted in a plot with 30 rows (1.2 m in length and inter-row distance of 20 cm). Thirty seeds per row and four rows per cultivar/line were sown. Huixianhong and Mingxian 169 were used as a susceptible control and border plants and were sown in every 10th row and around the plot. In spring, the seedlings of Huixianhong and Mingxian 169 were inoculated with a mixture of the 20 *Bgt* isolates collected from major wheat production regions of China. At full heading and milk stages, infection types (ITs) were rated using a 0–9 scale, where 0–4 was resistant, and 5–9 susceptible (Ma et al., 2018).

At the seedling stage, Jimai 23 and eight resistant donors with documented *Pm* genes (Supplementary Table S1) were tested for their reaction patterns to 42 *Bgt* isolates with different avirulence/virulence patterns. They were collected from major wheat production regions of China and isolated into single spore for virulence evaluation by Prof. Hongxing Xu (Supplementary Table S1). Each isolate was put in an independent transparent glass tube with layer of gauze to prevent cross-contamination among isolates. Five seeds of each genotype were sown in 128-cell rectangular trays in a growth chamber. The susceptible controls Huixianhong and Mingxian 169 were planted randomly in each tray. At the one leaf stage, the seedlings were inoculated with fresh conidiospores multiplied on Huixianhong seedlings, which were raised earlier and inoculated to provide a source of conidiophores for experimental inoculation. Then, the inoculated seedlings were incubated in a dark and independent chamber with high humidity at 18°C for 24 h. The trays were then placed in a climate incubator, set at a daily cycle of 14 h light at 22°C and 10 h of darkness at 18°C. ITs were surveyed when the spores were fully developed on the susceptible controls after about 10–14 days of inoculation using the 0–4 scale described by An et al. (2013), in which ITs 0, 0, 1, and 2 were regarded as resistant and ITs 3 and 4 as susceptible. Three repeats were tested using the same procedure.

To determine the inheritance of powdery mildew resistance in Jimai 23 and 22, all the *Bgt* isolates apart from those virulent to both Jimai 23 and 22 (Supplementary Table S1) were used to inoculate one-leaf seedlings of Jimai 23, Jimai 22, Tainong 18, and the F_1_, F_2_, and F_2__:__3_ progenies of Jimai 23 × Tainong 18 and Jimai 22 × Tainong 18 for genetic analysis. For the disease assessment of parents and F_1_ hybrids, 10 seeds were sown for inoculation with these isolates. For each F_2__:__3_ family, 30 plants were tested against these isolates. Goodness of fit was analyzed using a Chi-squared (χ^2^) test to assess deviations of the observed phenotypic data from theoretically expected segregation ratios using SPSS 16.0 software (SPSS Inc., Chicago, IL, United States) with a *p*-value level of 0.05.

### Preliminary Confirmation of *Pm* Gene(s) in Jimai 23

After genetic analysis, F_2__:__3_ families that were consistent with the ratio of monogenic segregation were selected for genotyping. Total genomic DNAs (gDNAs) of Jimai 23, Jimai 22, Tainong18, and the F_2__:__3_ families selected above were isolated from leaves after phenotypic evaluation using the TE-boiling method (He et al., 2017). Resistant and susceptible DNA bulks were constructed. For each, equal amounts of gDNA from either 10 homozygous-resistant or 10 homozygous-susceptible F_2__:__3_ families were pooled. Then, 50 simple sequence repeat (SSR) markers linked to documented *Pm* genes/alleles (Ma et al., 2016) were tested for polymorphisms between the parents and bulks. The polymorphic markers were then used to genotype the corresponding F_2__:__3_ families.

### Development of New Markers Using BSR-Seq

Total messenger RNA (mRNA) of Jimai 23, Tainong 18, and their F_2__:__3_ families inoculated with *Bgt* isolate YT01 (avirulent to Jimai 23) were extracted using the mirVana miRNA Isolation Kit (Ambion, Thermo Fisher Scientific Inc., Waltham, MA, United States) following the manufacturer’s protocol. The RNA integrity was assessed using the Agilent 2100 Bioanalyzer (Agilent Technologies, Santa Clara, CA, United States), and the samples with RNA integrity number (RIN) ≥ 7 were subjected to the subsequent construction of complementary DNA (cDNA) libraries. The cDNA libraries were constructed using TruSeq Stranded mRNA LTSample Prep Kit (Illumina, San Diego, CA, United States) according to the manufacturer’s instructions. After quality control of the cDNA libraries using the Agilent 2100 Bioanalyzer (Agilent Technologies, Santa Clara, CA, United States), the cDNA libraries were sequenced on the Illumina HiSeq sequencing platform (HiSeqTM 2500) by Biomarker Technologies Corporation (Beijing, China). After sequence assembly of the clean data with the reference genome of Chinese Spring (v1.0), SNP and small InDels in the targeted interval were obtained and used for marker development in BMK Cloud (developed by Biomarker Technologies Corporation).

### Genotyping of the Mapping Population and Map Construction

The developed markers were tested for polymorphisms between the parents and bulks. The resulting markers were genotyped on the F_2__:__3_ population of Jimai 23 × Tainong 18. Chi-squared (χ^2^) test was then used to assess deviations of the observed phenotypic data of the F_2__:__3_ families from theoretically expected segregation ratios for goodness of fit. The linkage map of the powdery mildew resistance gene(s) was constructed based on Lincoln et al. (1993) and Kosambi (1943) using the MAPMAKER 3.0 and the Kosambi function.

### Allelism Test

After the *Pm* gene in Jimai 23 was assigned to the *Pm2* interval, Jimai 23 was crossed with Wennong 14 and Liangxing 66, with documented *Pm2* alleles, to obtain F_2_ populations. The isolate YT01, that was avirulent to Jimai 23, Wennong 14, and Liangxing 66, was used to inoculate the F_2_ populations using Mingxian 169 and Huixianhong as susceptible controls. After the spores were fully developed on the susceptible controls, the number of resistant and susceptible plants of the F_2_ populations were counted to evaluate the allelic relationships between the *Pm* gene(s) in Jimai 23 and documented *Pm* genes on the same interval based on the ratio of resistant and susceptible F_2_ plants.

### Screening of Markers Available for MAS

To evaluate the utility of the markers of *Pm* gene(s) in Jimai 23 for MAS, the closely linked SSR markers were used to test the polymorphisms between Jimai 23 and 26 susceptible wheat cultivars from China (Supplementary Table S2). The markers that stably amplified polymorphic bands between Jimai 23 and the susceptible cultivars were regarded to be effective for MAS in these genetic backgrounds.

Jimai 23 was then crossed with some of the susceptible cultivars evaluated above to construct breeding populations. In the earlier generations, the resistant plants were selected for further self-pollination using the markers available for MAS. Meanwhile, the plants with poor agronomic performance in the field were eliminated. When homozygous-resistant plants were confirmed, they were mainly selected by agronomic performance in field. In the F_3_ or F_4_ generation, homozygous-resistant plants with suitable agronomic performance were planted in head rows. In the F_5_ generation, the best head rows were carried on to plot sowing. Finally, the stability of the breeding lines with superior agronomic and yield performance was confirmed once more by the markers and infection experiments.

## Results

### Comprehensive Traits of Jimai 23 in the Field

Compared with Jimai 22, Jimai 23 has comprehensively excellent traits. The GY of Jimai 23 was routinely higher than that of Jimai 22 although not significantly. More profoundly, the GI, SV, and STD of Jimai 23, which relate to flour quality, were significantly superior to those of Jimai 22, indicating that Jimai 23 has better processing quality than Jimai 22 (Table 1). This suggested that Jimai 23 has improved quality traits while still kept high yield potential of Jimai 22, making Jimai 23 an attractive wheat cultivar for both yield and quality.

**TABLE 1:** Agronomic, yield, and quality traits of wheat cultivars Jimai 23 and its parent Jimai 22.

Years	Cultivars	SNM (×10^4^)	KNS	TKW (g)	YM (kg)	BD (g/l)	GI	SV (ml)	STD (min)
2012–2013	Jimai 23	49.2 ± 0.9^a^	29.4 ± 0.4^a^	44.2 ± 1.2^a^	543.3 ± 9.8^a^	783.5 ± 20.3^a^	76.3 ± 2.3^a^	93.0 ± 2.1^a^	8.0 ± 0.3^a^
	Jimai 22	44.2 ± 1.1^b^	33.2 ± 1.1^b^	38.1 ± 1.4^b^	523.2 ± 15.1^a^	753.4 ± 22.3^b^	42.1 ± 1.8^b^	80.0 ± 1.3^b^	2.6 ± 0.1^b^
2013–2014	Jimai 23	45.5 ± 1.5^a^	33.1 ± 1.5^a^	49.0 ± 1.2^a^	618.3 ± 10.8^a^	811.0 ± 15.3^a^	64.0 ± 1.6^a^	37.2 ± 0.5^a^	4.6 ± 0.2^a^
	Jimai 22	45.5 ± 0.7^a^	35.6 ± 0.6^b^	44.6 ± 0.5^b^	597.2 ± 18.3^b^	800.3 ± 21.3^a^	30.0 ± 0.9^b^	26.7 ± 0.6^b^	3.1 ± 0.1^b^
2014–2015	Jimai 23	46.7 ± 2.1^a^	32.9 ± 1.2^a^	46.9 ± 1.9^a^	599.2 ± 10.3^a^	815.7 ± 12.2^a^	63.0 ± 1.3^a^	36.2 ± 0.9^a^	4.9 ± 0.2^a^
	Jimai 22	44.8 ± 2.3^a^	35.8 ± 1.3^b^	41.6 ± 1.5^b^	564.3 ± 7.8^b^	795.5 ± 10.3^a^	17.1 ± 0.5^b^	26.6 ± 0.2^b^	4.2 ± 0.0^b^
2015–2016	Jimai 23	43.4 ± 0.9^a^	33.4 ± 0.5^a^	49.0 ± 1.3^a^	617.7 ± 17.7^a^	798.8 ± 25.3^a^	62.0 ± 1.6^a^	37.0 ± 1.1^a^	8.5 ± 0.3^a^
	Jimai 22	41.3 ± 1.3^a^	36.0 ± 0.2^b^	45.7 ± 2.1^b^	597.5 ± 14.5^b^	788.4 ± 21.6^a^	40.3 ± 1.2^b^	32.0 ± 1.4^b^	3.1 ± 0.2^b^

*^*a,b*^Values in the same column followed by the same letter were not significantly different based on the test of least significant difference (LSD) at (*p* < 0.05). SNM, spike numbers per mu (1 ha = 15 mu); KNS, kernel numbers per spike; TKW, thousand kernel weight; YM, yield per mu (1 ha = 15 mu); BD, bulk density; GI, gluten index; SV, sedimentation value; STD, stable time of dough.*

### Evaluation of Powdery Mildew Resistance in Jimai 23

In the past three consecutive growing seasons (2017–2019), Jimai 23 showed high resistance to *Bgt* at the adult stage in the field, with an IT rating of 0–2, while Huixianhong was highly susceptible with an IT rating of 8–9. Compared with other resistance donors (Supplementary Table S1), Jimai 23 showed more satisfactory disease resistance. At the seedling stage, Jimai 23 was resistant to 39 of 42 *Bgt* isolates (92.9%) with diverse virulence profiles (Supplementary Table S1) and has a broader resistance spectrum than most of the documented resistant germplasms. This suggests that Jimai 23 is an elite resource for resistance breeding.

### Inheritance of Powdery Mildew Resistance in Jimai 23 and Its Genealogical Relationship With Its Parent Jimai 22

When inoculated with isolate YT01 that was avirulent to both Jimai 23 and Liangxing 99 (*Pm52*), the F_1_ plants of Jimai 23 × Tainong 18 and Jimai 22 × Tainong 18 were all resistant with an IT rating of 0, indicating the dominant nature of the resistance gene(s). The segregation ratio of Jimai 23 × Tainong 18 population was consistent with the ratio for monogenic segregation of a dominant gene, while the segregation ratio of Jimai 22 × Tainong 18 population was consistent with the independent separation of Mendelian law for two dominant genes (Table 2). While using isolates YT20 that was avirulent on Jimai 23 and virulent to Liangxing 99 (*Pm52*), the segregation ratios of Jimai 23 × Tainong 18 and Jimai 22 × Tainong 18 populations were both consistent with the ratio for monogenic segregation of a dominant gene (Table 2). Furthermore, using isolate YT03 that was avirulent to Liangxing 99 (*Pm52*) and virulent to Jimai 23, the segregation ratio of Jimai 22 × Tainong 18 population was consistent with the ratio for monogenic segregation of a dominant gene, while the while that of Jimai 23 × Tainong 18 population were all susceptible (Table 2). To confirm the segregation ratios of the populations when inoculated with other *Bgt* isolates that were avirulent to both Jimai 23 and Liangxing 99 (*Pm52*), three isolates YT05, YT23, and YT35, were selected to inoculate the F_2_ and F_2__:__3_ populations of Jimai 22 × Tainong 18 and Jimai 23 × Tainong 18. The results were consistent with those of YT01. These results suggested that the powdery mildew resistance in Jimai 23 may be controlled by a single dominant gene, whereas powdery mildew resistance in Jimai 22 is controlled by two dominant genes that fit independent separation of Mendelian law and that Jimai 23 most likely inherited one of the two *Pm* genes of Jimai 22.

**TABLE 2:** Segregation ratios of F_2_ and F_2__:__3_ generations of Jimai 23 × Tainong 18 and Jimai 22 × Tainong 18 following inoculation with different *Blumeria graminis* f. sp. *tritici* (*Bgt*) isolates at the seedling stage.

Crosses	YT01			YT20			YT03		
	Segregation ratio	χ^2^	*P*	Segregation ratio	χ^2^	*P*	Segregation ratio	χ^2^	*P*
Jimai 22 × Tainong 18 F_2_	(RR + Rr):rr = 234:14	χ^2^_15__:__1_ = 0.15	0.69	(RR + Rr):rr = 218:69	χ^2^_3__:__1_ = 00.14	0.70	(RR + Rr):rr = 201:71	χ^2^_3__:__1_ = 0.12	0.73
Jimai 22 × Tainong 18 F_2__:__3_	RR:Rr:rr = 109:112:12	χ^2^_7__:__8__:__1_ = 0.69	0.71	RR:Rr:rr = 58:118:62	χ^2^_1__:__2__:__1_ = 0.15	0.93	RR:Rr:rr = 59:117:65	χ^2^_1__:__2__:__1_ = 0.50	0.78
Jimai 23 × Tainong 18 F_2_	(RR + Rr):rr = 175:56	χ^2^_3__:__1_ = 0.07	0.79	(RR + Rr):rr = 199:61	χ^2^_3__:__1_ = 0.33	0.57	(RR + Rr):rr = 0:242	–	–
Jimai 23 × Tainong 18 F_2__:__3_	RR:Rr:rr = 47:103:50	χ^2^_1__:__2__:__1_ = 0.27	0.87	RR:Rr:rr = 57:115:55	χ^2^_1__:__2__:_ewan ko_1_ = 0.07	0.96	RR:Rr:rr = 0:0:225	–	–

*Values of χ^2^ for statistical significance at *p* < 0.05 are 3.84 (1 *df*) and 5.99 (2 *df*); RR, homozygous resistant; Rr, segregating; rr, homozygous susceptible.*

### Genetic Mapping of *PmJM23* and Its Genealogical Relationship With Its Parent Jimai 22

When inoculated with isolate YT20 that was virulent to Liangxing 99 (*Pm52*) and avirulent to Jimai 23, we detected a *Pm2*-linked marker polymorphism in both Jimai 23 × Tainong 18 and Jimai 22 × Tainong 18 populations using *Pm2*-linked marker *Cfd81*. When inoculated with isolate YT03 that was virulent to Jimai 23 and avirulent to Liangxing 99 (*Pm52*), we detected a *Pm52*-linked marker polymorphism in Jimai 22 × Tainong 18 population using *Pm52-*linked markers *Icssl326* and *Icssl795*, and when inoculated with isolate YT01 that was avirulent to both Jimai 23 and Liangxing 99 (*Pm52*), we detected *Pm2*-linked marker polymorphisms using five *Pm2*-linked markers (*Cfd81*, *Swgi069*, *Bwm20*, *Bwm21*, and *Bwm25*; Ma et al., 2018) and no *Pm52*-linked marker polymorphism using *Pm52-*linked markers *Icssl326* and *Icssl795* in Jimai 23 × Tainong 18 population. By combining the genealogical relationship between Jimai 22 and 23 with the marker detection results, we confirmed that Jimai 23 has a *Pm* gene nearby or in the *Pm2* interval, which we tentatively designate as *PmJM23.* We suggest that Jimai 22 has two *Pm* genes, one of which is *PmJM23* and another is the reported *Pm52* (Yin et al., 2009; Qu et al., 2019).

**FIGURE 1:**
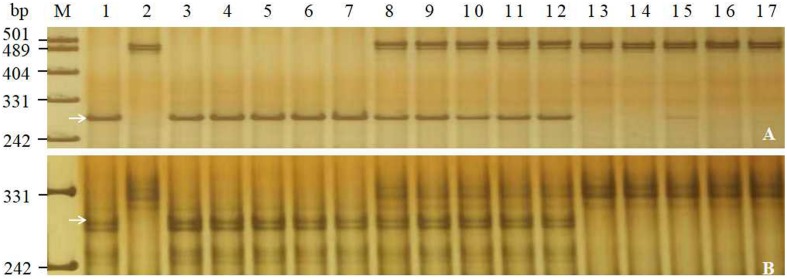
Amplification patterns of the selected markers *Bwm20* **(A)** and *YTU3004* **(B)** in genotyping Jimai 23, Tainong 18, and random selected F_2__:__3_ families of Jimai 23 × Tainong 18. Lanes M, pUC18 *Msp*I; lanes 1–2: parents Jimai 23 and Tainong 18; lanes 3–7: homozygous-resistant F_2__:__3_ families; lanes 8–12, heterozygous F_2__:__3_ families; lanes 13–17, homozygous susceptible F_2__:__3_ families. The white arrows indicate the 295 bp **(A)** and 290 bp **(B)** polymorphic bands in Jimai 23.

**FIGURE 2:**
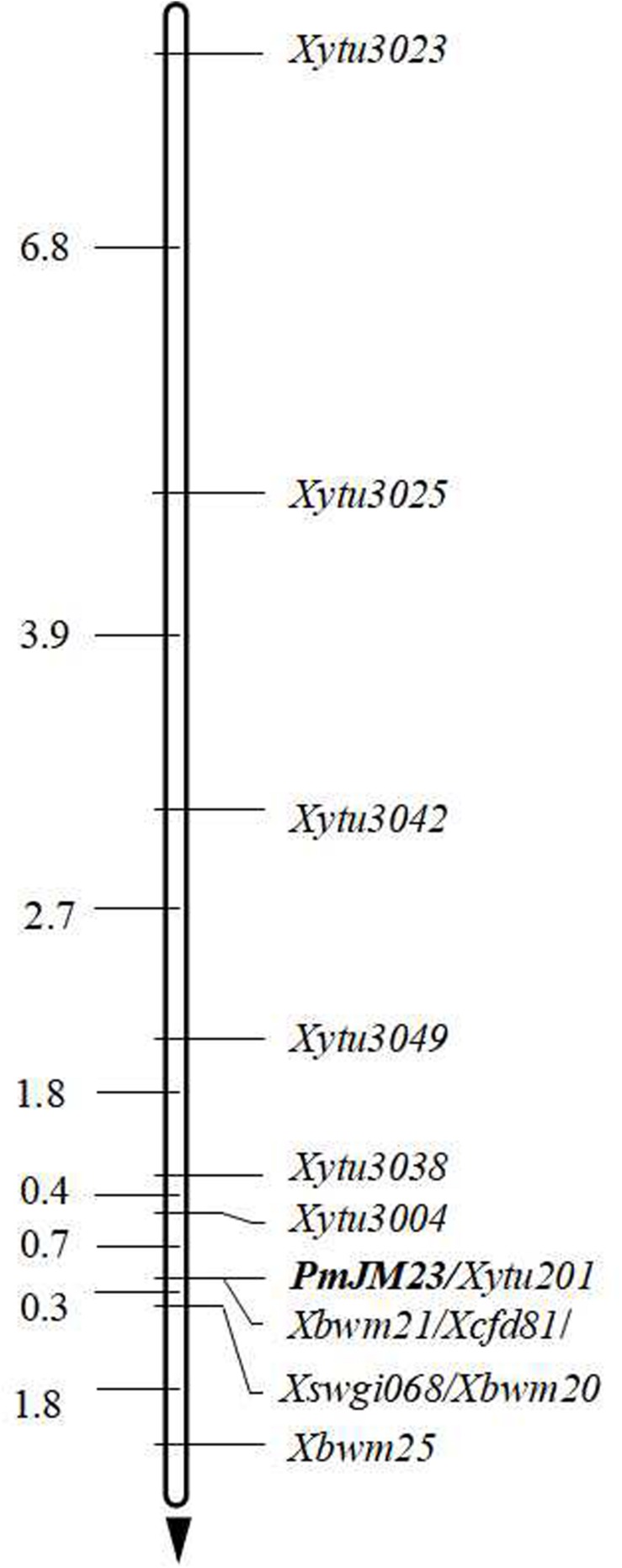
Linkage map of *PmJM23* using the F_2__:__3_ families of Jimai 23 × Tainong 18. Genetic distances in cm are shown to the left. *PmJM23* locus was set as the zero point. The black arrow points to the centromere.

To saturate the linkage map of the *PmJM23* interval, BSR-Seq was used to develop new markers linked to *PmJM23*. Using the SNPs and indels that distinguished or differed between resistant and susceptible parents and bulks in the targeted interval, 108 pairs of primers (Supplementary Table S3) were designed to screen polymorphic markers of *PmJM23* based on the SSR screening results in the interval (Supplementary Table S4). As a result, seven SSR markers (*YTU201*, *YTU3004*, *YTU3023*, *YTU3025*, *YTU3038*, *YTU3042*, and *YTU3049*) showed consistent polymorphisms between the parents and the resistant and susceptible bulks (Table 3). The markers were also linked with *PmJM23* after genotyping the F_2__:__3_ population of Jimai 23 and Tainong 18 (Figure 1 and Table 3). A linkage map was then constructed using newly developed markers and also combining the documented *Pm2* markers *Cfd81*, *Swgi068*, *Bwm20*, *Bwm21*, and *Bwm25* (Ma et al., 2018; Figure 2). *PmJM23* cosegregated with the markers *YTU201*, *Bwm21*, and *Cfd81* and was flanked by the markers *YTU3004* and *Swgi068/Bwm20* at genetic distances of 0.7 and 0.3 cm, respectively.

**TABLE 3:** New polymorphic markers developed using BSR-Seq for the powdery mildew resistance gene *PmJM23*.

Markers	Forward primer (5′–3′)	Reverse primer (5′–3′)	SSR_START	SSR_END	Product size (bp)	Marker type
*YTU201*	GAATTCTGTTGCACCCCTGG	GGAGCTACCCGACAATCACT	Unknown	Unknown	272	Co-dominant
*YTU3004*	GACATGCCACCACACCATAC	TGAGCTACCTAACCCAACCG	41091652	41091661	290	Co-dominant
*YTU3023*	GGGACTGCAGGTTGAATAATGG	CATGTGCTTTCCAACAATGCA	41797078	41797152	242	Co-dominant
*YTU3025*	TGGCGAAGTTCCATATTTTGC	GAGTGGGTTAGTAGGCAGGG	41870024	41870034	330	Co-dominant
*YTU3038*	AGCACTTGTATTCGAGCCCT	GGCATGGGTTCGTACTAGGT	42356673	42356688	390	Co-dominant
*YTU3042*	AACACATGTAGGCCCCGAAG	GCGACCCCATTTATTACCAGG	42602783	42602792	235	Co-dominant
*YTU3049*	GAGATTGTGTTTTCCATTGCGG	CAGGACACATCATCGCATCA	42914312	42914323	245	Co-dominant

### Allelic Test Between *PmJM23* and the Documented *Pm2* Alleles

Because *PmJM23* was assigned to the *Pm2* interval, the allelic relationship between *PmJM23* and documented *Pm2* alleles needed to be clarified using an allelic test. The phenotyping reactions of the 6,304 F_2_ plants between Jimai 23 and Liangxing 66 (*PmLX66*, allelic to *Pm2*), and 5,869 F_2_ plants between Jimai 23 and Wenong 14 (*PmW14*, allelic to *Pm2*) were surveyed. No susceptible plants were within all the tested F_2_ populations, suggesting that the *PmJM23* locus is most likely allelic to the *Pm2* locus.

### Comparisons of *PmJM23* and the Documented *Pm* Genes/Alleles at the *Pm2* Interval

When tested against 42 *Bgt* isolates, Jimai 23 showed a different response spectrum from other genotypes with documented *Pm* genes on chromosome arm 5DS, and especially from those of commercial cultivars (Supplementary Table S1). This information, combined with the allelic relationship between *PmJM23* and *Pm2*, demonstrated that *PmJM23* is most likely a new *Pm2* allele.

### Evaluation of Closely Linked Markers for MAS and Their Application in Breeding

**FIGURE 3:**
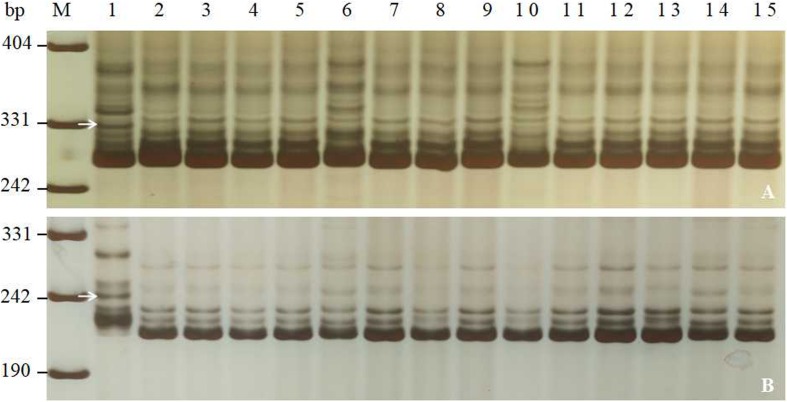
Amplification patterns of *PmJM23*-linked markers *YTU3025* **(A)** and *YTU3049* **(B)** in Jimai 23, Tainong 18, and selected wheat cultivars/breeding lines. M, DNA marker pUC18 *Msp*I; lanes 1 and 2, Jimai 23 and Tainong 18; lanes 3–15, wheat cultivars with sequential order of Huixianhong, Hanmai 13, Huaimai 0226, Xinong 979, Luchen 185, Jimai 268, Tainong 1014, Jinan 17, Liangxing 619, Shimai 15, Jimai 21, Jimai 20, and Shixin 633. The white arrows indicate the 330 bp **(A)** and 245 bp **(B)** polymorphic bands in Jimai 23.

Combined with comprehensive agronomic traits of Jimai 23, *PmJM23* is a valuable gene for *Pm* breeding. To transfer *PmJM23* to susceptible cultivars using MAS, the closely linked markers of *PmJM23* (*YTU201*, *YTU3004*, *YTU3038*, *YTU3049*, *Cfd81*, *Swgi068*, *Bwm20*, *Bwm21*, and *Bwm25*) were first tested for their suitability for MAS through detecting 26 susceptible cultivars. The results indicated that all the tested markers can amplify polymorphic bands between Jimai 23 and these tested cultivars, suggesting that once *PmJM23* is transferred into these genetic backgrounds through hybridization, these markers can be used to detect *PmJM23* (Figure 3 and Supplementary Table S2). In other words, these markers could be used in MAS for *PmJM23* in those genetic backgrounds.

**FIGURE 4:**
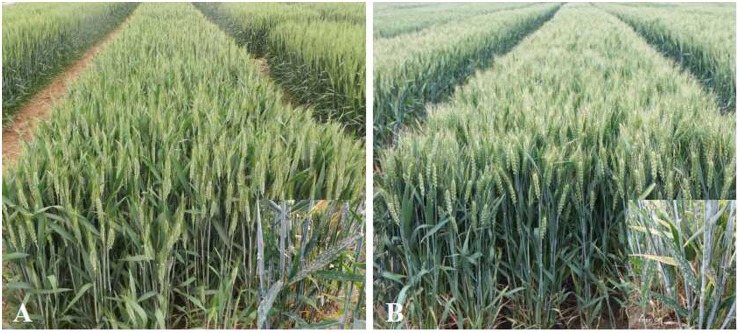
Field performance of Jimai 23 derived breeding line 19P084 **(A)** (Jimai 23 × Daimai 1503), 19P085 (Jimai 23 × Gaoyou 5766) **(B)**, that contained *PmJM23*. The left bottom in each figure are the susceptible controls Daimai 1503 and Gaoyou 5766.

To check the effectiveness of the markers available for MAS, Jimai 23 was crossed with a series of susceptible cultivars/breeding lines, including Gaoyou 5766, SH4300, Tainong 2419, 125574, Daimai 1503, Jimai 20, Hanmai 13, Yannong 19, and Luyuan 502. The F_2_ and F_3_ plants with linked marker alleles were selected using the corresponding markers. Combined with selecting for agronomic performance in field, head rows were planted in F_4_ generations. From the head rows, the hybridized combinations 19P084 (Jimai 23 × Daimai 1503), 19P085 (Jimai 23 × Gaoyou 5766), 19P086 (125574 × Jimai 23), 19P088 (Jimai 23 × SH4300), and 19P091 (Tainong 2419 × Jimai 23) with the best agronomic performance in the field were carried on to a field plot experiment. After comprehensive evaluation, two wheat breeding lines with superior agronomic performance in the field was selected. They were highly resistant to powdery mildew at both seedling and adult stages and have superior field performance (Figure 4). In the future, we expect these two lines to be evaluated in trials at both the provincial and national levels.

## Discussion

Jimai 23 is an elite wheat cultivar showing high resistance to powdery mildew during the whole growing season. The powdery mildew resistance in Jimai 23 is controlled by a single dominant gene, *PmJM23*. Using BSR-Seq, a series of new markers was developed and used to map *PmJM23* to a genetic interval, corresponding to the *Pm2* locus on chromosome arm 5DS. Resistance spectrum analysis demonstrated that *PmJM23* is a broad-spectrum *Pm* gene and hence is valuable for resistance breeding. A series of *Pm2* alleles have been identified in different wheat genotypes. Further genealogy tracing indicated that the *Pm2* alleles in landraces or breeding lines, such as *Pm2a* (Qiu et al., 2006), *Pm2b* (Ma et al., 2015a), and *Pm2c* (Xu et al., 2015), have unclear genealogy and that several wheat cultivars having the *Pm2* alleles, such as Liangxing 66 (genealogy: Ji91102/Jimai 19) with *PmLX66* (Sun Y.L. et al., 2015), Wennong 14 (genealogy: 84139//9215/876161) with *PmW14* (Sun Y.L. et al., 2015), Yingbo 700 (genealogy: Taigu sterility line/Jimai 19) with *PmYB* (Ma et al., 2015b), and Zhongmai 155 (genealogy: Jimai 19/Lumai 21) with *PmZ155* (Sun H.G. et al., 2015), have different genealogy with Jimai 23 (Jimai 22/Yumai 13) and Jimai 22 (genealogy: 935024/935106). Even resistance genes from the same original donor may also have variations diversified under selective pressure. Thus, these genotypes exhibited significantly different resistance spectra (Ma et al., 2018). However, whether a resistance gene can be used efficiently and rapidly in resistance breeding depends not only on the breadth of the resistance spectrum but also the comprehensive agronomic and quality characters of the donor. Wheat genotypes that exhibit resistance but which have poor or defective agronomic traits will slow down the breeding cycle because of the number of backcrosses required, which is not acceptable to breeders (Summers and Brown, 2013). Although many *Pm* genes, including *Pm2* alleles, have been identified, relatively few genes have been successfully used in modern breeding because of an imperfect balance between resistance and the agronomic traits of the resistance donors (Bie et al., 2015; Shah et al., 2018). In this study, Jimai 23 exhibited broader resistant spectrum than four of the six currently grown cultivars, and with isolate resistance similar to one cultivar (Supplementary Table S1). We found excellent correspondence between resistance and agronomic traits in Jimai 23. Moreover, *PmJM23* has a broader resistance spectrum than most of the resistant genes currently in production. We propose that Jimai 23 is an ideal resistance resource for wheat breeding.

In this study, *PmJM23* was mapped to the *Pm2* locus. Although this locus has been cloned by mutant chromosome sequencing (Sánchez-Martín et al., 2016) and analysis of the annotated genes in the mapping interval using the reference genome of Chinese Spring (Chen et al., 2019), no transgenic evidence was provided to confirm that the cloned sequence is the unique functional element conferring resistance to powdery mildew. Additionally, other reports showed that all the homologous sequences in different *Pm2* allele donors have the exactly the same sequence, yet these *Pm2* alleles exhibit significantly different resistance spectra that cannot be explained by the background differences of the resistant germplasms (Jin et al., 2018; Ma et al., 2018). In this study, the marker order across the *PmJM23* interval showed inversion and recombination phenomena (Figure 2 and Table 3). We speculate that the *Pm2* locus is most likely a complex locus that contains multiple elements conferring resistance to powdery mildew. To identify this resistance locus, map-based cloning using genomic information of the donor itself is imperative. In this study, we developed additional markers based on BSR-Seq that are located in the *Pm2* interval. These markers will accelerate map-based cloning of this locus and leading to the identification of the element(s) responding to powdery mildew.

Obviously, the complex *Pm2* locus has not been fully characterized, but this does not affect MAS of *PmJM23*. Using the markers newly developed in this work, the *PmJM23* locus as a breeding module can be efficiently transferred into susceptible cultivars. We showed that the newly developed markers are very suitable for MAS in different genetic backgrounds. Using these markers, we efficiently selected more than 20 breeding lines with greatly shortened breeding cycles and increased breeding efficiency. Five of these lines have superior agronomic traits and high resistance to powdery mildew. Thus, our study provides a successful model starting with evaluating the gene by resistance spectrum analysis, identifying the gene by genetic analysis, mapping the gene using closely linked markers and, finally, successfully transferring the gene by MAS.

## Conclusion

In this study, we identified and characterized a broad-spectrum *Pm* gene, *PmJM23*, in the elite cultivar Jimai 23 and successfully used it in MAS. This work is valuable as it provides the means of accelerating the utilization of *PmJM23* in breeding programs to control powdery mildew in wheat.

## Data Availability Statement

The raw data supporting the conclusions of this article will be made available by the authors, without undue reservation, to any qualified researcher.

## Author Contributions

PM and HH conceived the research. MJ, WD, WW, XZ, LW, and XL performed the experiments. HX, CL, and JS analyzed the data. RM, HL, JL, RH, and XW performed the MAS. PM wrote the manuscript. All authors read and approved the final manuscript.

## Conflict of Interest

The authors declare that the research was conducted in the absence of any commercial or financial relationships that could be construed as a potential conflict of interest.

## Footnotes

1 http://cb.natesc.gov.cn
